# By-Products of Heparin Production Provide a Diverse Source of Heparin-like and Heparan Sulfate Glycosaminoglycans

**DOI:** 10.1038/s41598-019-39093-6

**Published:** 2019-02-25

**Authors:** Sarah L. Taylor, John Hogwood, Wei Guo, Edwin A. Yates, Jeremy E. Turnbull

**Affiliations:** 1 Centre for Glycobiology, Department of Biochemistry, Institute of Integrative Biology, University of Liverpool, Liverpool, L69 7ZB UK; 2 National Institute for Biological Standards and Controls (NIBSC), Blanche Lane, South Mimms EN6 3GQ UK; 3 Suzhou Erye Pharmaceutical Co. Ltd, No. 2 Anmin Road, Dongqiao, Huangtai Town, Xiangcheng District, Suzhou, 215000 Jiangsu Province P.R. China; 4 Jieyang Runda Casing Co. Ltd., East side No. 1 Street, Jieyang High-tech Industrial Development Zone, Jieyang City, 515500 Guangdong Province P.R. China

## Abstract

Global production of pharmaceutical heparin (Hp) is increasing, and the production process from raw mucosal material results in large amounts of waste by-products. These contain lower sulfated Hp-like and heparan sulfate (HS), as well as other glycosaminoglycans, which are bioactive entities with pharmaceutical potential. Here we describe the first purification, structural and functional characterisation of Hp-like and HS polysaccharides from the four major by-product fractions of standard heparin production. Analysis of the by-products by disaccharide composition analysis and NMR demonstrated a range of structural characteristics which differentiate them from Hp (particularly reduced sulfation and sulfated disaccharide content), and that they are each distinct. Functional properties of the purified by-products varied, each displaying distinct anticoagulant profiles in different assays, and all exhibiting significantly lower global and specific inhibition of the coagulation pathway than Hp. The by-products retained the ability to promote cell proliferation via fibroblast growth factor receptor signalling, with only minor differences between them. These collective analyses indicate that they represent an untapped and economical source of structurally-diverse Hp-like and HS polysaccharides with the potential for enhancing future structure-activity studies and uncovering new biomedical applications of these important natural products.

## Introduction

Most of the globally important pharmaceutical, heparin (Hp), is extracted currently from porcine intestinal mucosa, although some is still obtained from bovine sources, mainly in South America. Extraction of the crude intestinal material yields approximately 0.8 Kg per animal which, following an extensive purification procedure provides typically 180–260 mg of Hp from each animal^1,2^. With the large number of animals (~1 billion per annum) required to meet the global demand for Hp, currently ~100 metric tonnes per annum^3^, many more tonnes of waste products are produced. Since the expanding global market for Hp is set to surpass 10 billion dollars by 2020, the increase in demand for raw porcine mucosal material for the isolation of the pharmaceutical product will also elicit an increase in the resulting by-products.

Owing to the specific size range of pharmaceutical Hp (average molecular weight typically ~12–15 kDa) and the sulfation selection criteria that are applied during the extraction and purification processes, less-sulfated or shorter Hp chains, as well as other GAGs including HS and chondroitin sulfate/dermatan sulfate (CS/DS), are discarded in the by-products. Previous work has demonstrated that HS can be obtained from crude Hp preparations^4^, and GAG preparations containing mixtures of Hp-like, HS and CD/DS polysaccharides such as sulodexide^5^ and danaparoid^6^ have also been prepared from mammalian mucosa for pharmaceutical use. Thus, Hp by-products could provide a wider potential source of novel Hp/HS GAGs containing a wealth of structural diversity and biological activities, at the same time making more efficient use of existing material without requiring the sacrifice of additional animals. Such materials could be utilised for research, or biotechnological and potentially medical purposes, where current supplies are scare and/or expensive.

The term HS was used originally to describe ‘heparin-like’ compounds that were extracted during the Hp manufacturing process^7,8^. Some authors suggested that Hp and HS were sufficiently similar to each other to enable them to be considered versions of the same molecule^9^, but other studies of HS from cell surface material suggested that they may be distinct^10^. Recent work, mainly concerning GAGs derived from marine organisms, however, has re-kindled this debate, since some of these marine-derived materials exhibit compositions that fall between prototypical Hp and HS, and also possess unexpected biological activities^11,12^. The possibility remains, therefore, that the original assertion may be correct and that the distinction between mammalian Hp and HS merely reflects a continuum of variations, with specific differences between the source tissues and species studied^9^.

Hp is a polydisperse linear polysaccharide whose detailed sequence is complex and variable between animals. In broad terms, Hp is composed of repeating 1–4 linked disaccharides comprising α L-iduronate/β D-glucuronate (IdoA/GlcA) and α D-glucosamine (GlcN). The highly anionic nature of Hp arises from the presence of carboxyl groups and extensive O- and N-sulfation; principally at positions 2 of iduronate and 6 and 2(N) of glucosamine but, much less frequently, also at position 3 of glucosamine. This provides Hp with an overall sulfation level of 1.4–2.5 sulfates per disaccharide unit, but most typically at the higher end of this range^9^. HS has underlying structural similarities to Hp, being composed of the same linkage types and basic monosaccharide units, but in different proportions and usually comprises much longer chains. HS possesses higher ratios of GlcA/IdoA and GlcNAc/GlcNS compared to Hp, and lower overall sulfation (typically 0.55–1.25 sulfates per disaccharide^7^ as well as better-defined domains of low sulfation (predominantly D-GlcA-D-GlcNAc) interspersed with shorter, more sulfated stretches^13,14^. It also differs in terms of the cells which produce it; almost all mammalian cells express HS, in contrast to Hp, which is produced by mast cells.

Hp is used widely as an anticoagulant in the clinic owing to its interaction with a number of serpins of the blood clotting cascade. Most notably, Hp potentiates the activity of antithrombin (ATIII) for subsequent inhibition of Factor Xa and IIa (thrombin). However, the nature of the interactions in the two cases are quite distinct. In the case of Factor Xa, a strong interaction between Hp and ATIII involves a pentasaccharide sequence, the most active form of which includes a central tri-sulfated glucosamine residue flanked by GlcA and IdoA2S. Interestingly, whilst a central 3-O-sulfated glucosamine (N-, 3-O- and 6-O-trisulfated glucosamine) is a key component in this known pentasaccharide sequence that binds strongly to antithrombin, the 3-O-sulfate modification to glucosamine also occurs in sequences which do not bind antithrombin with high affinity, and has been identified in HS from a variety of diverse sources with varying abundance^15–19^. Functionally, Hp can increase the affinity of ATIII for Factor Xa by ~1000-fold^20^. In contrast, the dual interaction between ATIII and Factor IIa requires a much longer heparin stretch, of at least 15 residues, but the sequence requirements are somewhat more relaxed; higher sulfation levels generally provide increased activity^20,21^. These differences led to the development of low molecular weight heparin (LMWH) with the ability to interact mainly with ATIII and culminated in development of the pentasaccharide drug Arixtra. Other direct interactions do occur between Hp and proteins of the blood clotting cascade, however, including Factors IX, XI and XII. While Hp has been optimised to some extent to provide a degree of selectivity in its interactions in the blood clotting cascade, it has also been identified as binding to hundreds of other proteins^22^. It is presumed that these interactions arise from the structural similarity between Hp and the ubiquitously expressed HS but, in most cases, interactions with HS have not been studied explicitly. In a recent sequence-based survey of 439 Hp binding proteins, no consensus sequence on the surfaces of these proteins for Hp binding emerged^23^ and, furthermore, the extent of true sequence specificity evident in this system has been questioned, leading to the suggestion that, ultimately, this class of compounds may provide a means of modulating protein networks more effectively than individual proteins^24–26^. In any case, it is evident that there are a considerable number of potential interactions that Hp and similar compounds could make, positioning HS-protein interactions as a crucial regulatory axis in biological processes. Importantly, this ultimately may be exploited for diverse pharmaceutical purposes. However, the further detailed study of these interactions requires access to diverse sources of HS structures.

The aim of the present work was to undertake the first systematic analysis of the structural and functional properties of Hp/HS GAGs in by-products that are discarded during the Hp manufacturing process. We characterised the four major by-product fractions in terms of their structural properties (such as disaccharide composition, sulfation and chain length), as well as biological properties (such as anticoagulant activity and activation of fibroblast growth factor signalling). The findings support the view that Hp by-products provide a rich resource of Hp-like/HS GAGs for structure-activity studies and have the potential to be exploited in future biomedical applications.

## Materials and Methods

### Purification and Recovery of Hp/HS By-Products

**Figure 1:**
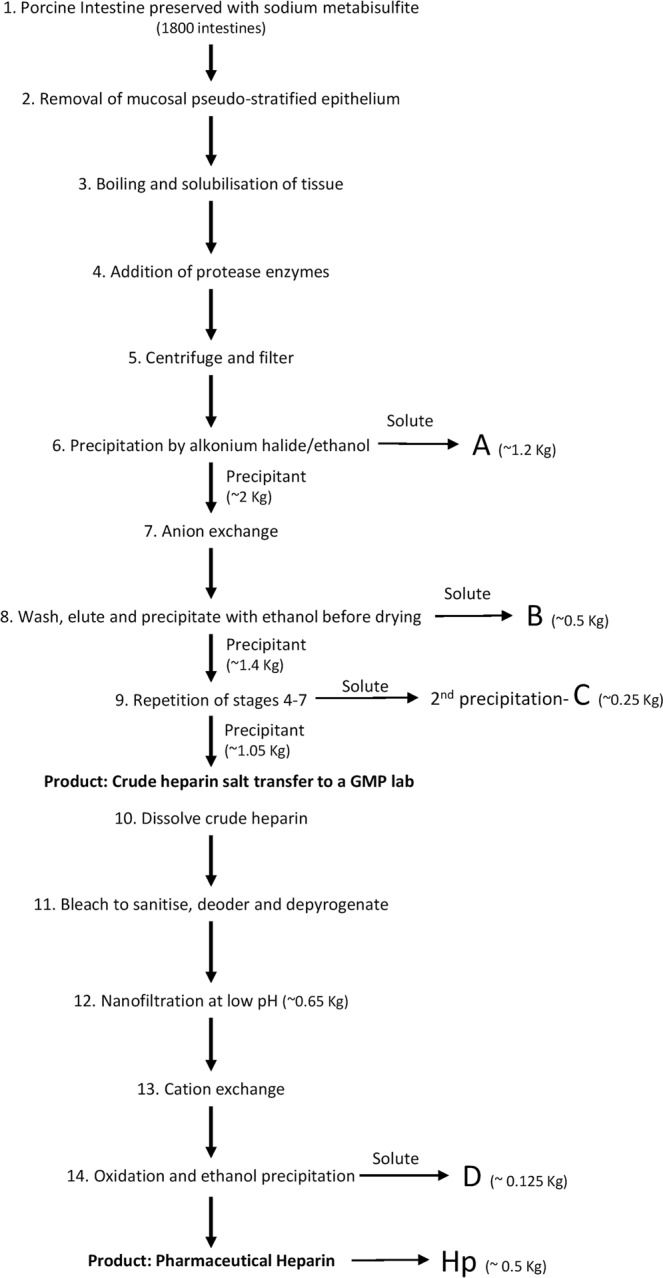
Flowchart depicting the major steps in the production of pharmaceutical Hp and the points at which the major four by-product fractions designated here as A-D were obtained. The production from porcine intestine at a slaughterhouse to final pharmaceutical Hp and LMWH, involves a clean-up process with multiple steps to ensure that all contaminants are removed, providing final products of pharmaceutical grade for human use. Estimates of typical yield quantities (kilograms) of the various intermediates, by-products and products from a production process starting with ~1800 intestines are as shown.

20 g of each crude by-product sample (Fig. 1; supplied by Chenzhong Biopharma, Shandong, China) was dissolved in 100 ml of chondroitinase buffer (100 mM tris acetate (Sigma, UK) pH 8.0). Samples were incubated until they had dissolved completely and had lost their viscosity. For chondroitinase digestion samples were incubated with 500 mU of chondroitin ABC-ase (Sigma-Aldrich, UK) overnight at 37 °C (all subsequent incubations are at 37 °C). After 24 hours, 20 ml of DNase buffer (10 mM tris acetate (pH 8.0), 25 mM magnesium chloride (Sigma, UK), 0.5 mM calcium chloride (Sigma, UK)) was added to the sample and in the presence of 500mU DNase and incubated for a further 4 hours. Further overnight digestions were conducted with 250 mU RNase in 20 ml of standard RNase buffer (10 mM tris acetate pH 8.0, 5 mM EDTA, 8 mM sodium acetate (Sigma, UK)) and subsequently 280 U of Pronase in 20 ml of buffer followed (10 mM tris acetate, 10 mM calcium acetate pH 7.0 (Sigma, UK)).

By-products were separated from the subsequent products of enzymatic digestion by weak anion exchange chromatography on diethylaminoethanol (DEAE) beads (batch preparation in 50 ml Falcon tubes – GE Healthcare, UK). 100 ml of 0.01 M phosphate buffered saline (PBS- Sigma, UK) and 100 ml of 0.3 M NaCl (ThermoFisher Scientific, UK) were used to clean the beads of all unbound non-HS products, HS was eluted with 2.0 M NaCl (100 ml). To remove the salt, the sample was dialyzed with 3.5 kDa cut off tubing (ThermoFisher Scientific, UK) over a 24-hour period with two 5 L changes of ddH_2_O.

### Separation of Purified Intact By-Products by Anion Exchange Chromatography

For analytical purposes, 200 µg of intact, clean crude heparin by-product was separated on a 1 ml Hitrap Q HP column (GE Healthcare, UK) over a 45 min programme set with two column volume washes of 0.5 M NaCl followed by a step gradient of 0.5, 1.0, 1.2, 1.4, 1.6, 2 M NaCl (10 ml of each) following a protocol similar to Griffin *et al*.^4^. Peaks were detected by following absorbance at 232 nm with the expectation that peaks would be visible across the range of NaCl concentrations and that more highly sulfated structures would be expected to elute at higher concentrations.

### Carbon-13 nuclear magnetic resonance spectroscopy

Each sample (20 mg) was dissolved in deuterated water (0.7 ml, >95% D, Sigma-Aldrich, UK), particulate matter was removed by benchtop centrifugation (5 mins) and the sample then transferred to a 5 mm NMR tube. One dimensional ^13^C spectra were recorded at 313 K on a Bruker 600 MHz spectrometer (Bruker, Germany) with 100 uM DSS (4,4-dimethyl-4-silapentane-1-sulfonic acid) as the external reference (0 ppm). Data sets for all spectra were processed in Mestrenova (Mestrelab, Spain). Signals were assigned according to the literature^27,28^.

### Compositional Analysis of By-Products by Digestion with Heparinase I, II and III and SAX-HPLC

Following clean-up, by-products were solubilised in heparinase buffer to make a solution of 10 mg/ml. 200 µg of by-products were digested with 2.5 mU of heparinase III, I and II (IBEX, Montreal, Canada) added sequentially at 2 hour intervals, with heparinase II left incubating at 37 °C overnight. Further digestion with fresh aliquots of all three heparinases was performed for 2 hours, to ensure samples were digested fully; no evidence of significant polymetric material was found by gel electrophoresis (data not shown). The products of digestion were separated according to charge on a strong anion exchange Propac PA1 column (4 mm diameter ×250 mm length- Dionex, UK), eluted with a linear gradient of 0–2 M NaCl, over 60 mins at 1 ml/min. Product peaks were detected by UV at 232 nm and samples were compared to 8 disaccharide standards of known structure (Dextra Labs, UK) also separated on the same column and under the same conditions.

### Molecular Weight Determination

Determination of weight average molecular weights for all samples was carried out using size exclusion chromatography on an Agilent GPC-50 (Agilent, Stockport, UK). The USP Heparin Sodium Molecular Weight Calibrant RS (US Pharmacopeial Convention, USA) was used to calibrate the system as previously described^29^.

### Antithrombin Dependent APTT, anti Xa and HCII anti-IIa/IIa assays

The test samples were assayed against the 6^th^ International Standard for Unfractionated Heparin (07/328, NIBSC, UK) using an automated coagulometer (ACL-TOP 550, Werfen Ltd, Warrington, UK). Dose response curves, consisting of at least three dilutions in replicate were assayed for each test material and the standard. Analysis of data was carried out in accordance with European Guidelines^30^ for biological materials using CombiStats 5.0 (European Directorate of the Quality of Medicine, France). Specific activities for each method were estimated using parallel line bioassay model in CombiStats. Plasma APTT, antithrombin dependent anti-IIa and anti-Xa and heparin cofactor II dependent anti-IIa assays were carried out as previously described^31^.

### BaF3 cell proliferation Assay

BaF3 (B-lymphoid) cells transfected with an FGFR1c receptor (gifted by Dave Ornitz, Washington State University, USA) were grown to confluence in a T75 flask (ThermoFisher Scientific, UK) in normal growth media (500 ml RPMI (Gibco, UK), 10% FBS (ThermoFisher, UK) and 50 µg/ml streptomycin (ThermoFisher Scientific, UK) supplemented with 2 ng/ml IL-3 (R&D systems, UK)). Cells were washed twice with normal growth medium (minus IL-3) by centrifugation and aspiration before being re-suspended in an appropriate amount of normal growth medium (1 × 10^4^/50 µl per well) and plated in triplicate in a 96-well plate (CoStar, UK). As controls for the experiment each of the following were added to the plate in 50 µl triplicates: 0.1 nM FGF2 (R&D Systems, UK) + assay media; 1 ng/ml IL-3 + assay media; 100 µg/ml Hp (Celsus, US) or by-products + assay media; and assay media alone. 50 µl of assay media containing 0.1 nM FGF2 was added to the plate in triplicate with the following concentrations of Hp or by-products: 1, 3, 10, 30 100 µg/ml.

Cells were incubated in test conditions at 37 °C in 5% CO_2_ for 72 hours before adding 5 µl of 5 mg/ml 3-(4,5-dimethylthiazol-2-YI)-2,5-diphenyltetrazolium bromide (MTT, Sigma, UK) followed by a further 4-hour incubation. In order to assess the colour change, which was used as a measure of cell proliferation, formazan is solubilised with  50 µl of 10% SDS/0.01 N HCL (Sigma-Aldrich, UK) per well and incubated overnight. Plates were read on a MultiScan Ex plate reader (ThermoFisher, UK) at 570 nm.

## Results

### Anion exchange chromatography demonstrates distinct sulfation profiles of the by-products

**Figure 2:**
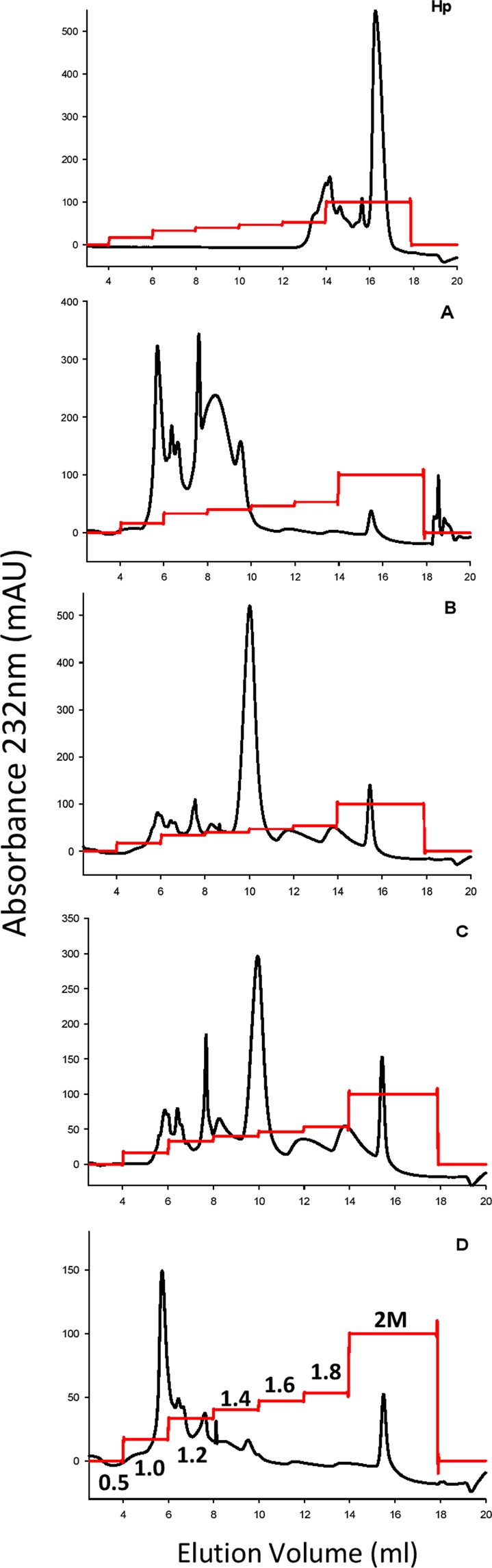
Strong anion-exchange chromatography of Hp and by-products. 200 µg of intact samples were separated by a step increase in concentration of NaCl (1.0, 1.2, 1.4, 1.6, 1.8 and 2 M, the red line is conductance and is an AKTA output with the scale relative to the maximum absorbance peak of the y-axis) at a flow rate of 0.5 ml/min. Samples were detected by UV at 232 nm.

We obtained a number of by-product fractions for our studies, and focused on the major side product fractions labelled A-D in Fig. 1. (in order of their stage of removal in the Hp production process). To initially characterise their anionic charge properties, we carried out a comparison by anion exchange chromatography (AEC) on a Hitrap Q HP column. Fraction A exhibited low levels of overall sulfation (Fig. 2). The majority of Fraction A was displaced from the AEC column prior at 1.0–1.2 M NaCl, although a small amount of residual material eluted at 2 M NaCl. Overall, this indicated that Fraction A may be predominantly low/moderately sulfated, but still contained some higher sulfated material. For Fractions B and C we observed a major peak at 1.4 M NaCl, with additional minor peaks eluted throughout the salt gradient at both lower and higher salt concentrations, including some 2 M material, which is present at higher levels in Fraction C compared to B. These fractions were removed at similar stages in the production of Hp, C being produced by a second round of filtration, anion exchange and precipitations (Fig. 1). Therefore, it may be relatively unsurprising that their AEC profiles showed very similar patterns indicating predominantly moderate sulfation, but also significant heterogeneity. Fraction D, which is the last by-product to be removed in the purification process, was distinct in that it contained the highest proportion of 2 M-eluting material, but also a significant proportion eluting at only moderate (1–1.2 M) NaCl. These results are consistent with Fraction D comprising the highest proportion of highest proportion of highly sulfated Hp-like structures. Overall, these results indicated that each by-product was distinct in terms of the spread of sulfated species, and that a broad correlation exists between increase overall sulfation of the by-products with their later stage of removal in the production process.

### Carbon 13 NMR indicates differences in by-products compared to Hp

**Figure 3:**
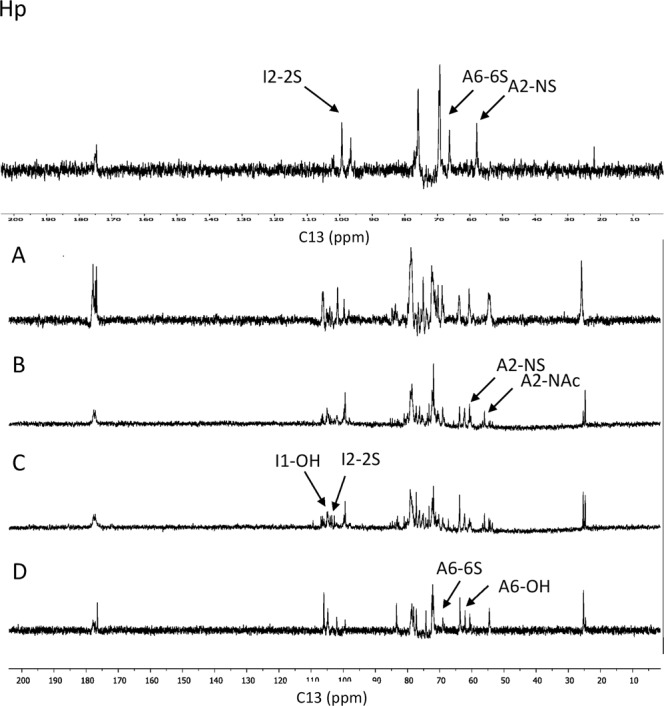
One dimensional ^13^C NMR of Hp and by-products. (**A**) NMR spectra of 20 mg of samples in D_2_O were recorded at 313 K on a Bruker 600 MHz spectrometer with DSS as the external reference (0 ppm). Signals were assigned according to Yates *et al*.^23^. Sulfation at glucosamine position 6 is denoted 'A6-6S', at position 2 of glucosamine, A2-NS and at position 2 of iduronate, as I2-2S.

To further elucidate the structure of the by-products carbon-13 nuclear magnetic resonance spectroscopy (^13^C NMR) is a powerful and non-destructive method with which to explore the structure of Hp^27,28^. Using ^13^C NMR to compare Hp and each of the by-products, it was evident that Hp was substantially more sulfated than the by-product materials (Fig. 3), supporting the SAEC chromatography results (Fig. 2). In addition, Fraction D appeared to the most similar in terms of its relatively high level of sulfation, consistent with the AEC data (Fig. 2). However, the ratios of N- and O- sulfation of B also showed a comparable level of sulfation to D, with higher levels of N-sulfation (1.3 – B and D, Table 1) and both B and D also contained similar amounts of 2-O sulfation (0.7). Fraction B appears to contain more 6-O sulfation than D (0.9 vs 0.7). Fraction A contained intermediate sulfation, with the ratios of all 3 sulfation patterns being similar to each other (N- 0.8, 2- 0.9, 6- 0.7). Fraction C was the least sulfated sample, with all sulfate positions in similar abundance (N- 0.7, 2- 0.8, 6- 0.8). In previous work, N-unsubstituted glucosamine residues have been identified in some commercial porcine mucosal HS samples^32^ but, here, we observed no significant characteristic signals at 96 ppm for such residues in any of the by-products, or Hp (Fig. 3). Overall the NMR data support the view that each by-product is distinct, but also indicated that the rank order of the by-products in terms of proportions of sulfations at the key N, 2 and 6 positions was D > B > A > C.

**Table 1:** Ratios of peak height of sulfated/unsulfated residues at A-6, A-2, and I-2 for Hp and by-products. 2-O sulfation of iduronic acid is most clearly identifiable by the chemical shift of carbon 1 at 102 ppm.

Carbon Sample	A6 (69 ppm)	A2 (57 ppm)	I1 (102 ppm)
**Sulfated/unsulfated**			
Hp	3	2.7	2.6
A	0.7	0.8	0.9
B	0.9	1.3	0.7
C	0.7	0.7	0.8
D	0.7	1.3	0.7

N and 6-O sulfation are best identified by changes in signal of carbon 2 and 6 of N-acetylglucosamine at 57 and 69 ppm, respectively. 6-O-sulfation in glucosamine (evident in the ^13^C NMR spectrum at 69 ppm) is referred to as ‘A6’, N-sulfation in glucosamine (58 ppm) as ‘A2’ and 2-O-sulfation (monitored by the signal for C-1 in iduronate residues (102 ppm) as ‘I1’.

### Disaccharide analysis indicates that by-products are structurally distinct from Hp and from each other

**Figure 4:**
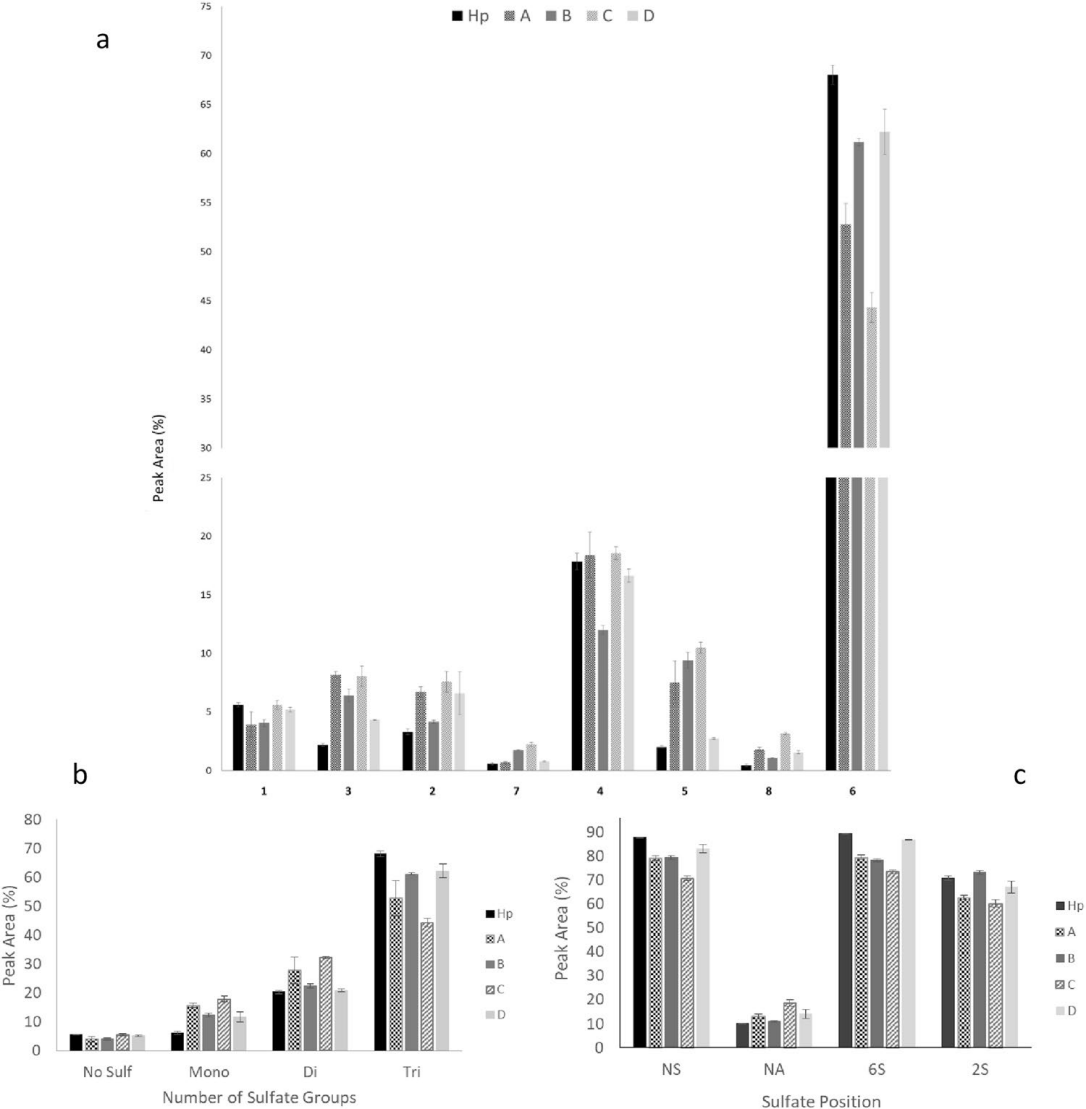
Disaccharide analysis of Hp and by-products. (**a**) Following enzymatic digestion with heparinase I, II and III samples were separated on a Dionex PA-1 column over a 0–2 M NaCl gradient, products were identified by UV at 232 nm. A set of 8 known structures were also run under the same conditions and these were used to identify peaks from the samples (Table 2). Percentage peak areas were calculated for each sample, along with the SEM and multiple t-tests comparing heparin to the other samples (p = 0.05, n = 3). (**b**) Analysis of sulfation levels and types from disaccharide composition data. From the same data used in 4a, percentage peak areas for each sample were grouped into categories to determine other structural compositions. Percentage peak areas for no sulfates^1^, mono- (3, 2, 4) di- (5, 7, 8) and tri- (6) were calculated, with mean and SEM. Multiple comparison t-tests (p = 0.05, n = 3) between all sample to Hp were carried out. (**c**) Percentage peak areas were also grouped to examine the positions of sulfation; NS^3–6^, NA^1,2,7,8^, 2S [7, 5, 8. 6] and 6S^2,4,6,8^ were calculated, with mean and SEM. Multiple comparison t-test (p = 0.05, n = 3) comparing all samples to Hp.

The samples were compared to Hp at the disaccharide level. Hp is characterised as having low levels of the unsulfated disaccharide UA-GlcNAc (<5%) and high levels of the trisulfated disaccharide UA(2S)-GlcNS(6S) (typically >60–70% in PMH), giving rise to high average sulfation (~2.5 sulfates per disaccharide). In order to compare disaccharide composition of the by-products to Hp, samples were subjected to exhaustive lyase digestion and the products of the digestion were separated by strong anion exchange (SAX)-HPLC on a Propac PA1 column, using a 0–2 M NaCl gradient, with material detected by UV absorbance at 232 nm. Compositional analysis at the disaccharide level demonstrated significant differences between each of the by-products, and also to Hp as a comparator (Fig. 4a). Fractions B and D contained the highest amount of trisulfated disaccharide (~60 and ~62% respectively), whereas Hp contained ~68%. Fractions A and C showed statistically significant reduction in the proportion of trisulfated ∆UA(2S)-GlcNS(6S) disaccharide which is characteristically the most abundant disaccharide in Hp (Table 2). Fractions B and C showed the largest differences at this level when compared to Hp. Fraction C exhibited low levels of sulfation overall, with significant decreases in the trisulfated disaccharide and concomitant increase in all mono- and di-sulfated structures, except for ∆UA-GlcNS(6S). There was also a significant decrease in the unsulfated disaccharide ∆UA-GlcNAc and, similar to fraction C, a significant increase in all mono- and disulfated disaccharides, except for ∆UA-GlcNS(6S).

**Table 2:** The structure of 8 standard disaccharides used for HS/Hp comparison. These 8 standards have defined structures and elute in a reproducible order.

Peak (in elution order)	Structure
1	Δ-UA-GlcNAc
3	Δ-UA-GlcNS
2	Δ-UA-GlcNAc(6S)
7	Δ-UA(2S)-GlcNAc
4	Δ-UA-GlcNS(6S)
5	Δ-UA(2S)-GlcNS
8	Δ-UA(2S)-GlcNAc(6S)
6	Δ-UA(2S)-GlcNS(6S)

Considerable variation was evident in the proportions of the N-sulfated disaccharides ∆UA-GlcNS and ∆UA(2S)-GlcNAc(6S) in by-products from different stages of the production process. Fractions A and D exhibited the least significant differences from Hp, however, both showed a further difference in content of ∆UA-GlcNAc(6S) (p = 0.004) and ∆UA(2S)-GlcNS (p = 0.003).

Whilst there are minimal differences between the by-products and Hp in terms of unsulfated disaccharide (Fraction B has a significant decrease, p = 0.01 – Fig. 4a and Table 2), there are more notable changes in the levels of other disaccharides. All by-products showed a significant increase in monosulfated disaccharides compared to Hp, A, p = 0.0005, B, p = 0.001, C, p = 0.0005 and D, p = 0.04. Fraction C also had increased proportions of disulfated structures (p = 0.0001) and exhibited a decrease in the trisulfated disaccharide (Table 2).

All by-products showed a lower proportion of disaccharides containing at least one sulfate group, compared to Hp. Structures containing N sulfates are significantly less well-represented in Fractions B (p = 0.0003), C (p < 0.0001) and D (p = 0.05). 6-O sulfation is also significantly reduced in Fractions B (p = 0.0003), C (p < 0.0001) and D (p = 0.0005), and finally 2-O sulfation in Fraction C was significantly lower than in Hp (p = 0.004). This establishes that all by-products are significantly less sulfated than Hp and the production process progressively selects for retention of more sulfated species at each purification stage. Overall the data confirmed that each by-product sample has a distinct sulfation profile, and that they all differ from Hp.

### The by-products differ in average sulfation levels, and fraction C is the least sulfated

**Figure 5:**
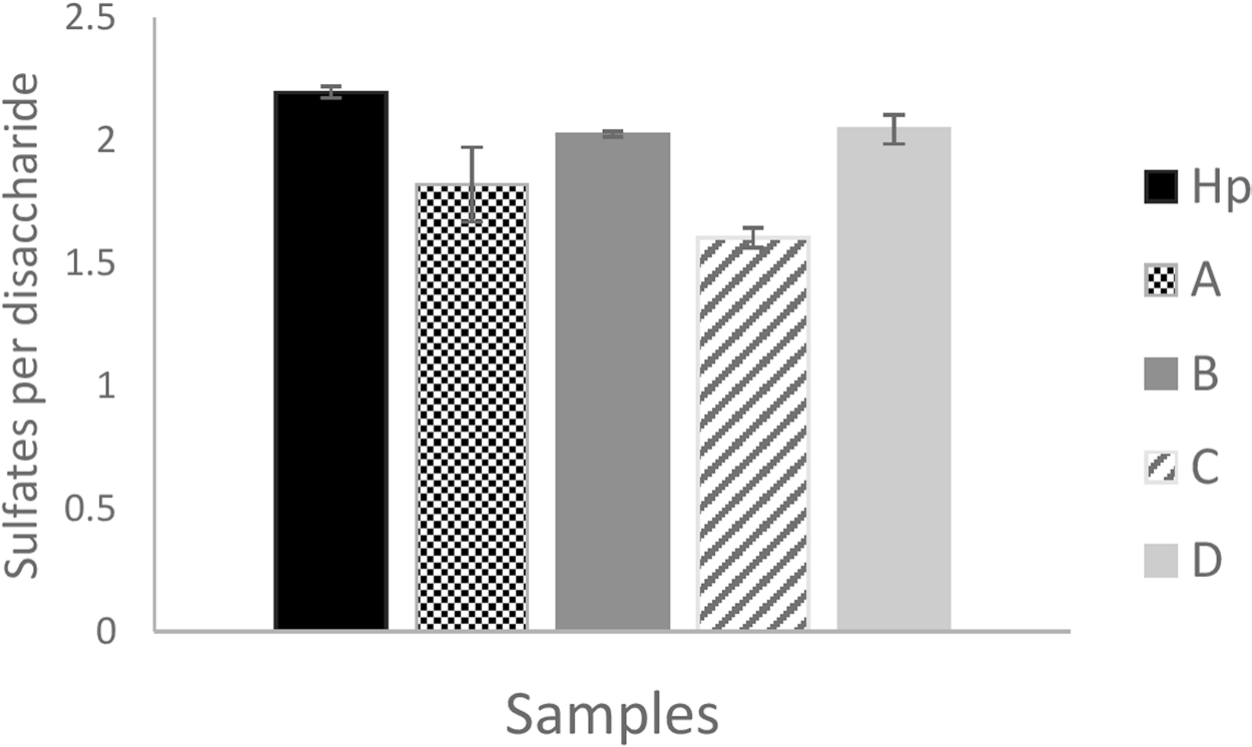
Average number of sulfates per disaccharide in Hp and by-products. Values were calculated from each sample triplicate values from Fig. 4a. The SEM was calculated and all samples were compared to Hp via multiple t-tests (p = 0.05, n = 3).

Average sulfation levels were compared based on the disaccharide composition data from Fig. 4a; as mentioned previously Hp is characterised by being highly sulfated containing 2.0 or more sulfates per disaccharide, and consistent with this we observed a value of ~2.2 (Fig. 5). Fraction B and D had a similar level of sulfation to Hp, with 2.1 and 2.0 sulfates per disaccharide, whereas Fraction A was lower at ~1.8. Fraction C was clearly the least sulfated (average sulfation of 1.6 per disaccharide unit). This data, based on disaccharide composition, indicates that the rank order of the by-products in terms of average sulfation levels was D > B > A > C, consistent with the data obtained by NMR (Fig. 3).

### The by-products are more polydisperse than Hp

**Figure 6:**
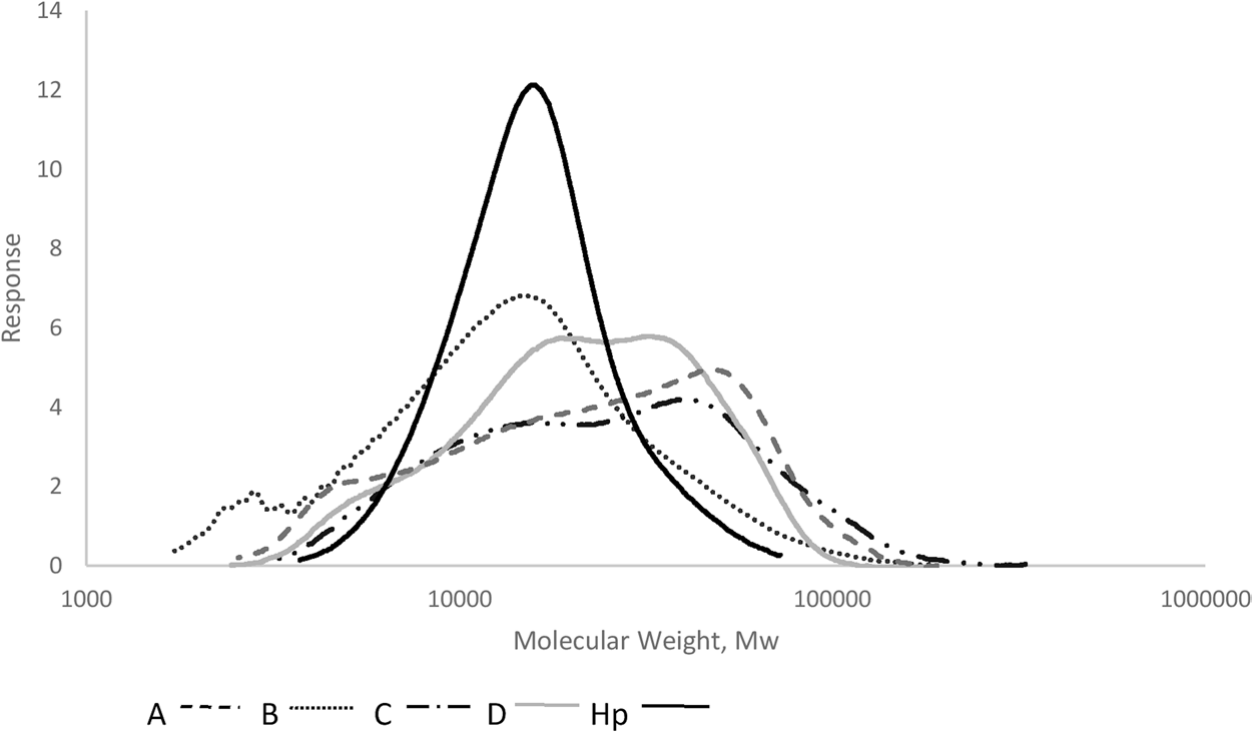
Molecular weight determination of the by-products by gel permeation chromatography against a Hp calibrate. Average molecular weight (Mw) suggested no correlation with purity, however, the polydispersity (Pd = Mw/Mn)) was reduced up to and including the removal of Hp. The loss of sample below 100% in the detected range was due to the resolution range residing between 4 and above 40 kDa (Hp – 99.7%, A – 94.3%, B – 90.3%, C – 94.4%, D – 98.1%). *Mn – number average molecular weight. Average molecular weight (kDa) – Hp, 16.6, A, 30.6, B, 18.7, C, 33.5, D, 26.3. Pd – Hp, 1.2, A, 2.2, B, 2.0, C, 2.1, D, 1.7.

The molecular weight of Hp (~7–20 kDa) is distinct to that of HS; the latter containing a greater proportion of longer chains (20–110 kDa)^33^. Gel permeation chromatography analysis against a broad standard calibrant allowed both weight and number average molecular weights (Mw and Mn) to be determined for the fractions, as well as their distributions within defined ranges; Mw 4–16, 17–28, 29–39 and >40 kDa. The polydispersity, (Mw/Mn), was also calculated from data obtained for Fig. 6. All the by-products had higher Mw than Hp, in the range ~17–34 kDa (see Fig. 6), and there is considerable variation in both Mw and Mn as the purification progresses. The polydispersity, a measure of the extent of chain length variation in these linear polymers, broadly decreases as the Hp purification process proceeds, and all the by-product fractions displayed greater polydispersity than Hp. Any increases in molecular weight may result from selective losses from the parental material (disproportionate losses of different molecular weights). Overall this analysis of the by-products by gel permeation chromatography suggests that molecular weight criterion is not strongly selected during the purification process for Hp.

### The by-products have distinct anticoagulant activity profiles and reduced potency compared to Hp

As a first step in characterising potential functional differences, we carried out assays of various anticoagulant activities of the by-products, in comparison to Hp as a positive control. We measured both the activity of the intrinsic and extrinsic anticoagulant cascade. All of the by-products were found to have greatly reduced ability to inhibit coagulation compared to Hp. The APTT assay, which measures the intrinsic and common pathways, including factors II, V, X and fibrinogen, indicated that all by-product fractions were 2.7–9.2 fold less able to inhibit coagulation than Hp, with D having the highest and A the lowest activity (Table 3). Specific HCII, Anti-Xa and IIa activities were in broad agreement with the global anticoagulant activity of these samples reflected in the APTT assay. All fractions were less able than Hp to inhibit these factors, in the same order as in the APTT assay (rank order D > B > C > A), with C and A by far the least able to inhibit HCII (A, 16.7 fold, C, 13.6 fold) FXa (A, 9.2, fold C, 9 fold,) and FIIa (A, 15.6 fold, C, 11.2 fold,) compared to Hp. Thus, all the by-products had low anticoagulant activity and were distinct from Hp. But in addition, each also had a distinct profile of relative activities in the different assays compared to each other.

**Table 3:** Anticoagulant activity of the 6^th^ international standard of Hp and Fractions A-D in global APTT and specific Xa, IIa and HCII IIa assays.

Sample	Plasma APTT	AT anti-IIa	AT anti-Xa	HCII anti-IIa
**Specific Activity – IU/mg (95% CL)**				
A	22.7 (21.9–23.5)	13.4 (12.7–14.1)	23.2 (21.8–24.7)	14.4 (13.2–15.6)
B	70.8 (68.4–73.4)	57.4 (54.5–60.5)	60.1 (56.4–64.0)	54.3 (50.0–58.9)
C	22.8 (27.8–29.8)	18.6 (17.7–19.6)	23.8 (22.3–25.3)	17.7 (16.2–19.2)
D	77.0 (74.4–79.8)	72.9 (66.5–79.9)	79.1 (74.2–84.3)	63.0 (58.1–68.4)
Hp	209 (191–229)	209 (196–223)	214 (207–221)	241 (223–261)

### All the by-products retain the ability to activate FGF2 signalling in a dose-dependent manner

**Figure 7:**
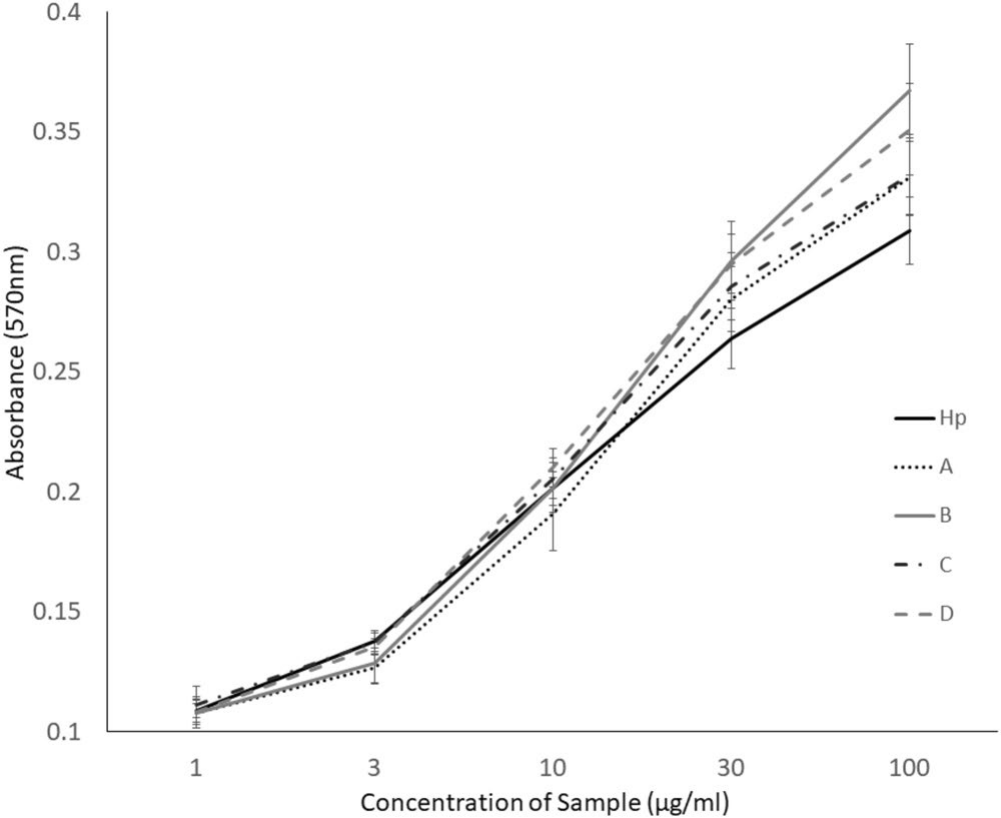
Proliferation of BaF3-1c cells was induced by Hp or by-products in response to FGF2. Cells were incubated in the presence of 0.1 nM FGF2 and a range of by-product samples or Hp control (1–100 µg/ml) for 72 hours. Proliferation was determined by MTT assay, reading the absorbance at 570 nm (n = triplicate of 3 biological repeats). The mean and standard error for each sample was calculated and multiple t-tests (p = 0.05) were carried out for all repeats, at all concentrations and samples were compared to Hp. P-values with a significant increase over Hp – 100 µg/ml A, 0.002, B, <0.0001, C, 0.002, D, <0.0001, 30 µg/ml A, 0.005, B, <0.0001, C, 0.0006, D, <0.0001, 10 µg/ml D, 0.01. P-values with a significant decrease below Hp – 3 µg/ml A, <0.0001, B, 0.002.

As a further test of the relative bioactivities of the by-products, we also assessed their ability to activate cellular signalling by fibroblast growth factor (FGF2). B-lymphoid cells are dependent on the successful activation of a transfected FGFR1c or IL-3 receptor. In the absence of either IL-3 or FGF1/2 these cells are unable to induce signalling required for their survival and therefore undergo apoptosis. HS and Hp have been shown to be essential for the successful complex formation by the growth factor and receptor^34^ and therefore this assay identifies those saccharides which are able to induce successful signalling and therefore survival and proliferation of the cells. From an initial assessment, all by-product samples stimulated survival and proliferation in a concentration dependent manner, similar to that of Hp (Fig. 7). There was minimal impact on cell proliferation at the lowest concentration. However, a large increase in proliferation was seen at concentrations between 3 and 30 µg/ml. All by-product samples showed significant changes in the induction of proliferation at several concentrations, compared to Hp (p < 0.05), particularly at 30 and 100 µg/ml (Fig. 7). Samples B and D showed substantially more activity at higher concentrations compared to Hp along the concentration curve (Fraction B, 100, 30, 3, 0.1 and 0.03 µg/ml; Fraction D, 100, 30, 10, 0.3 and 0.1 µg/ml – Fig. 7). This suggests that they were better able to activate the FGF2/R1c pathway compared to Hp. By-product A, whilst exhibiting a significant increase in proliferation over Hp at several concentrations (3 µg/ml and to a lesser extent at 100 and 30 µg/ml), appeared to have, overall, comparable activity to Hp. C was the least able to induce proliferation, although higher proliferation levels than Hp were observed at 3 concentrations (100, 30 and 0.3 µg/ml).

## Discussion

The production of Hp for the pharmaceutical market is extensive, generating ~100 tonnes/annum of active Hp pharmaceutical ingredient^3^. The process for the isolation of what is defined as Hp from porcine mucosa selects highly sulfated structures of low polydispersity in chain size, with high anticoagulant potency and reduced heterogeneity compared to the GAGs in the raw material. In contrast, we show here for the first time that the four major side-fractions, produced as waste materials of this production process, possess lower sulfation and greater heterogeneity, increased chain size polydispersity, and lower anticoagulant activity relative to Hp. They are thus quite distinct from Hp in terms of both structure and activity, and represent a relatively untapped resource for production of Hp-like and HS polysaccharides. Although some mixed GAG preparations such as sulodexide, a mixture of LMWH and dermatan sulfate;^5^ and danaparoid, a mixture of HS, DS and CS^35^; have been prepared from crude mammalian mucosa extracts for pharmaceutical use^5,6^ details of their structural characterisation have been largely lacking until recently^36^. HS GAGs from crude porcine mucosal Hp preparations have been previously described^4^, and recent work has also highlighted the need for careful structural characterisation of such preparations to avoid potential production artefacts such as de-N-sulfated glucosamine^32^.

Here we undertook a systematic purification and analysis of the four major by-product fractions from the standard production process for Hp. We focussed on the Hp-like/HS GAGs contained in these fractions. To the best of our knowledge this is the first such analysis of these side fractions, which represent a valuable potential resource for this class of natural products. We established that, in terms of disaccharide composition, they are all clearly unique from each other and from Hp. The by-products differ most significantly in the proportions of disaccharides Δ-UA-GlcNAc(6S), Δ-UA(2S)-GlcNS and Δ-UA(2S)-GlcNS(6S). Average sulfation levels (~1.6 to 2.1) were, as expected, lower than found in Hp (2.2). Although higher average sulfation broadly correlated with later stage of removal in the production process (in rank order D > B > A > C), Fraction C was an exception since it had the lowest sulfation. Thus the correlation was not absolute, suggesting that the purification processes may not fractionate solely on the basis of average charge, but also patterns of sulfation. It should be noted that Fraction C was obtained by a repeat of the purification step that was used to obtain B, and we would expect to see lower sulfated material that was not removed during the first round of this purification step.

Importantly, we did not observe detectable amounts of N-unsubstituted glucosamine (GlcN) in our preparations. Such moieties have recently been observed at high levels in some commercial preparations of porcine mucosal HS^32^, indicating that care is needed to subject final purified batches to detailed structural analysis such as those we have undertaken (especially rigorous NMR analysis), to ensure the integrity of the material in its natural form.

In terms of chain size, all the by-products were larger than Hp, with average sizes ranging from ~19 kDa for the smallest (Fraction B) to ~34 kDa for the largest (Fraction C), compared to ~17 kDa for Hp. Regarding polydispersity, we noted that the by-products were broadly similar (ranging from 1.7 to 2.2), all of them displaying much higher polydispersity than Hp (1.2; see Fig. 6). Our data suggest that the Hp production process progressively reduces the polydispersity of the starting material to generate a more homogenous final Hp product. The polydispersity of the finished product is known to be manufacture dependent, with the average polydispersity of Hp on the market being 1.3^23^, in good agreement with our current analysis. The reduction in polydispersity is also coupled with selection for increasing sulfation throughout the process (summarized in Fig. 6).

Importantly, we also demonstrated that side-fractions can be sub-fractionated by AEC (Fig. 2), indicating heterogeneity of these HS by-products as observed previously in HS samples purified from raw Hp^4^. The patterns of separation by AEC clearly demonstrated that each fraction is distinct from each other, as well as from Hp. Although Fractions B & C showed quite a similar range of fractions, they were present in different proportions (Fig. 2). Interestingly, the AEC data indicated that all materials contained some highly sulfated Hp-like material eluting at 1.8–2.0 M NaCl. Overall these heterogeneous patterns are significant as they demonstrate the presence of distinct sub-populations of HS. Furthermore, the AEC method we used is readily scalable and could be used for preparative production of these fractions, which could be exploited in future studies to generate a wider range of HS sub-preparations with variant sulfation levels and patterns. Such preparations could be exploited effectively in binding assays or bioassays to examine their differential protein interactions and bioactivities, and could also be exploited to develop more divergent oligosaccharide libraries via fractionation methods dependent on depolymerisation, size and sulfation^37^.

As anticipated, the by-products we studied also displayed clear diversity in their functional properties. Firstly, the anticoagulant activity of the by-products is significantly reduced compared to Hp, similar to that observed for HS sub-fractions from crude Hp studied by Griffin *et al*.^4^. Furthermore, we found that all the by-products showed a large reduction in global and specific anticoagulant activity, increased activity correlating with the fractions removed later in the Hp production process (Table 3). Secondly, we found that all the by-products retain substantial biological activity comparable to that of Hp for promoting activation of FGF signalling. This was demonstrated in an assay of cell signalling through FGF receptor 1c and FGF-2 (Fig. 7). Interestingly, side-products B and D showed a concentration dependent increase in cell proliferation, and both were significantly more active at low and high concentrations than Hp. We speculate that further sub-fractionation of the individual purified by-products, such as by AEC (Fig. 2), will be useful in future studies to separate different HS sub-species that will likely display variant activities in FGF signalling and other biological assays of HS-dependent mechanisms.

Analysis of the by-products further supports the view that Hp production succeeds in efficiently isolating the majority of Hp chains possessing high anticoagulant activity, and having high sulfation and low chain polydispersity. Clearly they represent a characteristic sub-set of Hp/HS fractions contained within raw Hp. Conversely, this results in the production of by-products with increased polydispersity, lower sulfation, and higher structural heterogeneity, which retain significant and divergent biological activities. It will be of significant interest in future studies to explore the 3-O-sulfate content of the by-products to investigate how this rare but important O-sulfate modification relates to anticoagulant and other bioactivities. Overall, these under-utilised waste products could therefore provide an economic and valuable source of useful bioactive natural products. These could be exploited to accelerate future structure-activity studies via oligosaccharide libraries^33^ and also to reveal new biomedical applications of these natural products as novel biomaterials^38^ or therapeutics.
